# Left ventricle remodeling by CMR in treated patients with primary aldosteronism and primary systemic arterial hypertension

**DOI:** 10.1371/journal.pone.0316140

**Published:** 2024-12-23

**Authors:** Carolina S. Reiser, Antonildes N. Assuncao, Jose A. B. Araujo-Filho, Roberto N. Dantas, Luiz A. Bortolotto, Jose R. Parga-Filho

**Affiliations:** Instituto do Coração (InCor), Hospital das Clinicas HCFMUSP, Faculdade de Medicina, Universidade de Sao Paulo, Sao Paulo, São Paulo, Brazil; Scuola Superiore Sant’Anna, ITALY

## Abstract

**Background:**

Increased cardiac after load and multiple non-hemodynamic stimuli implicate in adverse left ventricular remodeling (LVR). This is particularly identifiable in treatment-resistant and secondary hypertension contexts, like primary hyperaldosteronism (PA), however little data exists on post-treatment residual LVR in these individuals.

**Methods:**

Cardiac magnetic resonance (CMR) with T1 mapping were performed in 14 patients with treated PA matched with 15 treated patients with primary hypertension (PH) and 15 healthy individuals. Blood pressure (BP) control was defined as < 140 x 90mmHg.

**Results:**

Treated PA and PH patients had similar indexed left ventricular, extracellular matrix and intracellular masses (respectively 68 ± 12g/m2, 17 ± 3g/m2 and 52 ± 10g/m2 for PA vs 63 ± 18g/m2, 16 ± 5g/m2 and 47 ± 14g/m2 for PH, p > 0.05 for all), that were significantly higher than normal individuals (47 ± 8g/m2, 11 ± 2g/m2 and 36 ± 6g/m2, respectively, p < 0.05 for all). Patients with uncontrolled BP exhibited greater cardiomyocyte hypertrophy than those controlled (55 ± 11 g/m2 vs 43 ± 11 g/m2, p = 0.01), regardless of the cause of hypertension. PH individuals had strong correlations between BP measurements and LVR parameters of the CMR, while in PA correlations were weaker.

**Conclusions:**

In treated patients with PA and PH, CMR detected similar residual tissue LVR in both groups. Uncontrolled BP was more related to the observed LVR than to the etiology of hypertension. BP levels were more strongly correlated to CMR LVR parameters in PH than PA patients.

## Introduction

Hypertension remains a major preventable cause of cardiovascular disease [1]. The prevalence of blood pressure (BP) control has decreased in several population surveys around the world, despite the considerable progress in strategies to achieve this goal [2–4]. Factors such as the perceived cardiovascular disease risk, treatment adherence, the physicians’ willingness to a more aggressive approach and a true resistant hypertension have been reported as some of the barriers [3, 4].

The increased wall stress due to sustained higher hemodynamic afterload in patients with uncontrolled BP is partially responsible for an adverse left ventricular remodeling (LVR), a complex process driven also by multiple nonmechanical stimuli [5], and is strongly linked with long-term cardiovascular outcomes [5–7]. This is particularly true in the clinical scenario of difficult-to-control hypertension, where secondary causes must be investigated, being primary aldosteronism (PA) a relevant etiology [1]. Aldosterone excess seems to amplify hemodynamic effects of hypertension, both by direct activating specific receptors and molecular signal pathways, resulting in exacerbated hypertrophic response, as well as inducing chronic inflammation and left ventricle (LV) fibrosis [8].

Cardiovascular magnetic resonance (CMR) can provide excellent non-invasive mass, volume and systolic function quantification, allowing global access of myocardial geometric maladaptation [5, 9, 10]. CMR has, also, unique ability to provide tissue characterization, using late gadolinium enhancement (LGE) to detect focal fibrosis and tissue mapping techniques to quantify diffuse involvement in compartmental levels [9, 11].

There is a lack of comprehensive data on myocardial tissue changes in asymptomatic patients with difficult-to-treat hypertension, particularly in patients with PA. This study aims to investigate the role of CMR on the assessment of subclinical disease in treated patients with PA and primary hypertension (PH) followed in a tertiary center specialized in hypertensive disease.

## Methods

### Ethics statement

The study was founded by the Fundação de Amparo à Pesquisa do Estado de São Paulo (FAPESP), process number 2014/05650-6. The funders had no role in study design, data collection and analysis, decision to publish, or preparation of the manuscript. Written informed consent was obtained from all patients, according to local Ethics Committee—Comissão Científica do InCor do HCFMUSP (SDC 4013/13/138) and Comitȇ de Ética CAPPesq.

### Study design and population

This prospective observational study enrolled 15 consecutive PA patients on medical treatment, 10 without and 5 with adrenal adenoma (2 that declined surgery and 3 previously resected but that remained hypertensive despite treatment), and 17 PH patients with matched age and sex. Their CMR imaging was performed from 21/10/2014 to 28/08/2020. All patients were ≥ 18 years-old and followed at the Hypertension Unit of the Instituto do Coração (InCor) of the University of São Paulo, a large tertiary hospital. Patients who refused to sign the informed consent form, those with a clinical or imaging diagnosis of other cardiomyopathies and patients with atrial fibrillation (potentially associated with incorrect estimates of T1 relaxation time) were not included in this study. Patients with contraindications to gadolinium-enhanced magnetic resonance imaging, as listed below, were also excluded: pregnancy, allergy to contrast, estimated renal creatinine clearance less than 30 mL/min, patients with pacemakers or implantable devices incompatible with magnetic resonance imaging. From the included hypertensive patients, 3 were later excluded from the analyses due to poor imaging quality, 1 from the PA group (with adenoma previously resected) and 2 from the PH group.

PH and PA diagnoses were made according to recommended guidelines [12]. In brief, PA patients were screened, after a washout period of interfering hypertensive medications, using aldosterone to renin ratio (positive if ≥ 30), with posterior confirmation, when applicable, through saline infusion test (positive if post-infusion plasma aldosterone > 5ng/dL) [12]. Of note, PH patients at our Institution are usually primary care referrals due to difficult-to-treat hypertension.

We also used age and gender matching based on a nearest-neighbor matching algorithm to compared the data from our cohort of patients with those of anonymized 15 healthy age and gender-matched controls (HC), derived from a series of normal individuals from our local database [13, 14]. The analyses of the HC group data, as well as the data from both hypertensive groups, was anonymized and performed together from 01/04/2021 to 10/07/21.

### CMR imaging protocol

Scans were performed with a 1.5T CMR equipment (Philips Achieva, Best, Netherlands). Cine-resonance, T1 mapping and LGE images were obtained as previously described [15]. T1 mapping used a Modified Look-Locker Inversion Recovery (MOLLI) sequence, 3(3)3(3)5 sampling pattern, with the following parameters: thickness of 10 mm, field of view 300x300mm, matrix ACQ 152x150, flip angle 40, minimum inversion time (TI) of 60ms and TI increment of 150ms. MOLLI images in midventricular short axis were acquired prior and 20 minutes after an intravenous bolus of 0.2 mmol per kilogram of body weight of gadolinium-based contrast (Dotarem^®^, Guerbet Aulnay-Sous-Bois, France). T2 mapping was not part of the study protocol.

### Image post-processing

Imaging post-processing of cine, T1 mapping and LGE images were performed according to position statements of the Society of the Cardiovascular Magnetic Resonance [16]. All CMR images were analyzed together using a dedicated software (Medis Medical Imaging Systems, Leiden, Netherlands) by a trained radiologist blinded to clinical data, with >5 years of cardiovascular imaging experience. Left ventricle hypertrophy (LVH) was considered when indexed LV mass index (LVMI) was higher than 81g/m2 for men and 61g/m2 for women [17]. LGE was measured using semiautomatic quantification considering a threshold of 5-SD.

**Fig 1:**
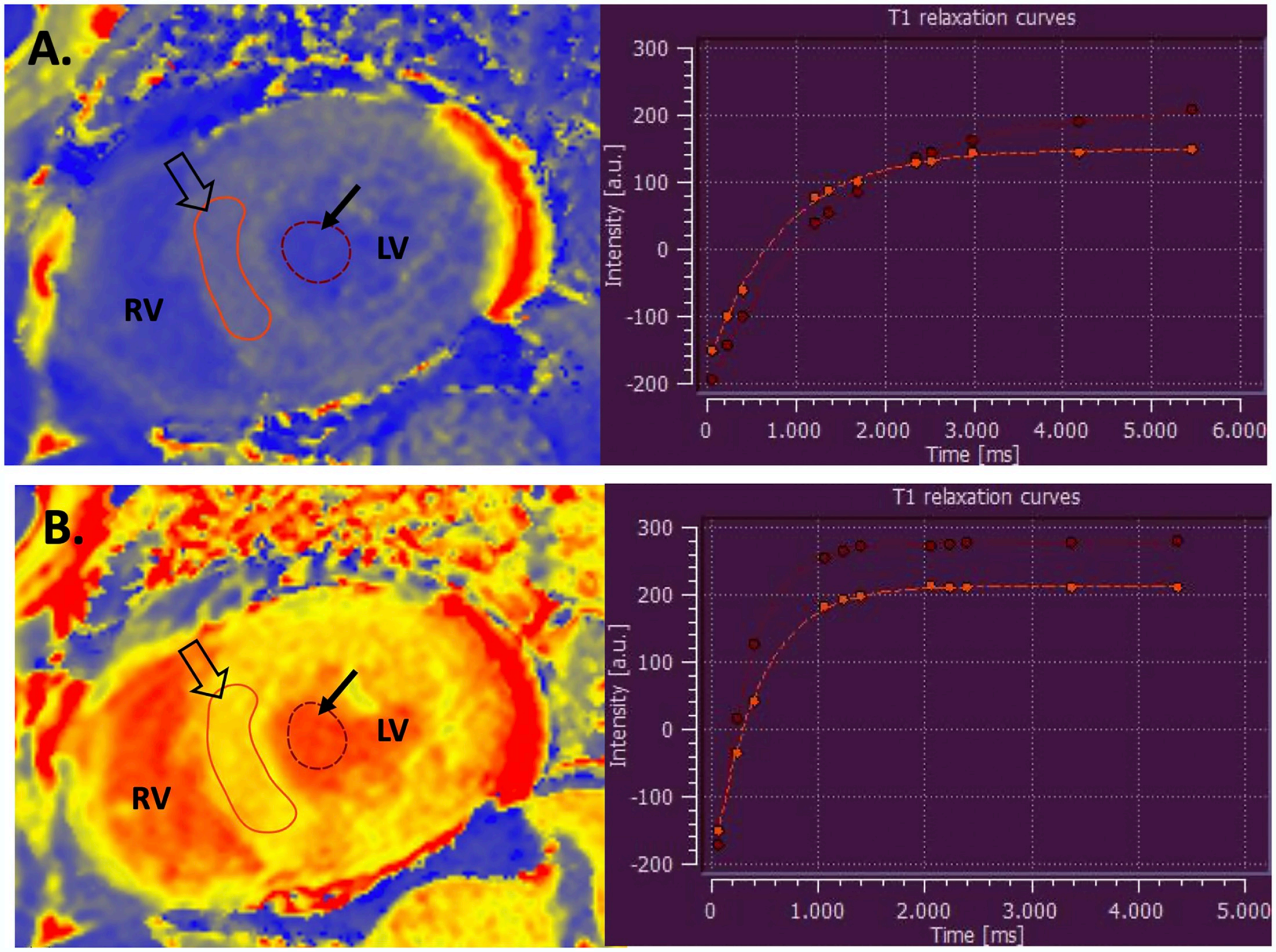
Demonstration of ROI placement for T1 mapping. Midventricular short axis plane pre (A) and post (B) gadolinium infusion, with respective T1 relaxation curves. The arrows show cavity (blood) ROI and open arrows show septal (myocardial) ROI. ROI: region of interest, RV: right ventricle, LV: left ventricle.

T1 estimation of remote myocardial was performed using single ROI drawn on midventricular short axis septum (Fig 1). T1 values were reported only for segments without LGE. Extracellular volume (ECV) was then calculated using the partition coefficient (λ) and contemporaneous hematocrit (HCT), with a median time interval from CMR acquisition of 9 days. Residual of ECV (i.e., 1 − ECV) was registered as intracellular volume (ICV). LV extracellular mass (ECMI) was defined as ECMI = indexed LVM × ECV (g), whereas intracellular mass (ICMI) was defined as ICMI = indexed LVM − ECMI (g).

### BP measurement

Hypertensive patients had two BP levels measured in office visits at the Hypertension Unit after five minutes of seated rest, with 30-second intervals. Hypertensive patients had a median of 7 medical visits. The mean of all measurements was used to define the systolic (SBP) and diastolic (DBP) BP levels. Controlled BP was defined as SBP < 140 and DBP <90 mmHg across all visits [4, 18].

### Statistical analysis

Data are expressed as mean ± standard deviation, [interquartile range] and frequency (percentage). Normality assumption was assessed graphically using QQ plots. For continuous variables, differences between the three groups were calculated using one-way analysis of variance or Kruskal-Wallis as appropriate. Hommel’s method, a stepwise multiple testing correction technique, was applied to control the family-wise error rate and adjust p-values for multiple comparisons [19]. To assess differences in CMR parameters between patients with and without controlled BP, t-test (or rank-sum) was used accordingly. Correlations between CMR features and BP levels also performed by Spearman test. We investigated whether hypertension etiology modifies the relationship between BP measurements versus CMR features by using an interaction term in a linear regression model. Finally, we built a regression model to investigate the independent effect of controlled BP and PA on LVMI and ICMI.

All statistical analyses were performed using R version 4.1.2 (R Foundation for Statistical Computing, Vienna, Austria) and a p-value<0.05 was considered statistically significant.

## Results

### Participant characteristics

Clinical characteristics of the 15 healthy controls and 29 hypertensive patients (14 with PA and 15 with PH) are reported in Table 1. Groups were well matched with respect to age and sex, with no significant differences. The prevalence of cardiovascular risk factors (diabetes, dyslipidemia and smoking history) did not significantly differ between PA and PH groups. Most patients (62%) had more than 10 years of hypertensive disease. Patients with PA presented higher baseline SBP (at diagnosis) when compared with those with PH. Yet, next to CMR, BP levels and prevalence of patients with controlled BP were comparable between the two groups. The number of antihypertensive agents did not differ significantly between PA and PH patients (p > 0.05). All patients in the PA group were treated with three or more antihypertensive agents, including 100% receiving angiotensin-converting enzyme inhibitors (ACEi) or angiotensin receptor blockers (ARB). In comparison, 71% of PH patients were on three or more agents, with 93% receiving ACEi or ARB. As expected, the use of mineralocorticoid receptor antagonists (MRA) was significantly higher in the PA group compared to the PH group.

**Table 1:** Clinical characteristics across groups.

	HC	PH	PA
	(n = 15)	(n = 15)	(n = 14)
Age [years]	48 ± 13	54 ± 13	56 ± 10
Male, no. (%)	8 (53)	9 (60)	6 (43)
BSA [m^2^]	1.81 ± 0.19	1.97 ± 0.16	1.93 ± 0.20
BMI [kg/m^2^]	27 ± 5	32 ± 4^†^	31 ± 6^‡^
Aldosterone [ng/dL]	-	9,85 [5,58–13,12]	23,55 [20,85–35,40]*
Plasma renin activity [ng/dL/h]	-	3,08 [0,49–6,19]	0,33 [0,16–0,48]*
A/R	-	13,80 [1,03–26,85]	93,65 [49,35–131,88]*
Serum creatinine [mg/dL)	0.93 [0.86–0.98]	0.98 [0.84–1.04]	1.01 [0.88–1.30]
Hematocrit [%]	44 ± 2	43 ± 3	42 ± 4
Heart rate [bpm]	72 ± 12	68 ± 10	70 ± 10
Diabetes, no. (%)	-	6 (40)	6 (43)
Dyslipidemia, no. (%)	-	5 (33)	9 (64)
Smoking history, no. (%)	-	7 (47)	4 (29)
Time from diagnosis > 10years, no. (%)	-	8 (53)	10 (71)
Baseline SBP [mmHg]	-	159 ± 16	177 ± 23*
Baseline DBP [mmHg]	-	102 ± 12	107 ± 15
CMR SBP, mean (SD)	118 ± 6	143 ± 25^†^	146 ± 30^‡^
CMR DBP, mean (SD)	66 ± 7	92 ± 16 ^†^	88 ± 16 ^‡^
Mean SBP, mean (SD)	-	150 ± 23	153 ± 27
Mean DBP, mean (SD)	-	93 ± 13	92 ± 11
Controlled BP, n (%)	-	6 (40)	6 (43)
BP-lowering agents, no. (%)			
0	-	0 (0)	0 (0)
1	-	1 (7)	0 (0)
2	-	3 (21)	0 (0)
≥3	-	10 (71)	14 (100)
ACEi/ARB, no. (%)	-	14 (93)	14 (100)
MRA, no. (%)	-	6 (43)	12 (86)*

HC: healthy controls, PH: primary hypertension, PA: primary aldosteronism, BSA: body surface area, BMI: body mass index, A/R: aldosterone/renin ratio; SBP: systolic blood pressure, DBP: diastolic blood pressure, CMR SBP and DBP: BP levels by the time CRM exam was performed, ACEi: angiotensin converting enzyme inhibitor, ARB: angiotensin receptor antagonist, MRA: mineralocorticoid receptor antagonist.

* p<0.05 PA versus PH,

^†^ p<0.05 PH versus HC,

^‡^ p<0.05 PA versus HC.

### CMR LV parameters

Hypertensive groups (PH and PA) presented similar LV volumes and function when compared to the control group. As showed in Table 2, patients with PA and PH had significantly higher LVMI, diastolic anteroseptal and inferolateral LV thickness than controls. Of note, only 3/15 patients with PH had LVH, versus 7/14 patients with PA (p = 0.02), and there was a tendency to a higher mass/volume ratio in PA than PH (0.93 ± 0.18g/mL and 0.82 ± 0.17g/mL, p = 0.057).

**Table 2:** Parameters of LV morphology, function, and tissue characterization by CMR across groups.

	HC	PH	PA
	(n = 15)	(n = 15)	(n = 14)
**LV morphology and function**			
LVEDVI [ml/m2]	74 ± 9	76 ± 13	74 ± 11
LVESVI [ml/m2]	27 ± 5	29 ± 9	26 ± 5
LVMI [g/m2]	47 ± 8	63 ± 18 ^†^	68 ± 12 ^‡^
M/V [g/ml]	0.64 ± 0.07	0.82 ± 0.17 ^†^	0.93 ± 0.18 ^‡^
LV septal thickness [mm]	9,0 ± 1,4	11.9 ± 2.4^†^	12.4 ± 2.3^‡^
LV posterior thickness [mm]	7.6 ± 1.4	9.4 ± 1.6 ^†^	9.7 ± 2.0^‡^
LVEF [%]	63 ± 5	62 ± 6	65 ± 4
**Tissue Characterization**			
LGE+, no. (%)	-	7 (47)	7 (50)
LGE [% total LV Mass]	-	2.8 ± 1.3	2.5 ± 2.3
Native T1 [ms]	1039 ± 27	1033 ± 25	1041 ± 48
Post-contrast T1 [ms]	534 ± 34	508 ± 49	508 ± 33
ECV [%]	24 ± 2	26 ± 5	25 ± 3
ICV [%]	76 ± 2	74 ± 5	75 ± 3
ECMI [g/m2]	11 ± 2	16 ± 5 ^†^	17 ± 3 ^‡^
ICMI [g/m2]	36 ± 6	47 ± 14 ^†^	52 ± 10 ^‡^
ECMI/ICMI	0.32 ± 0.04	0.35 ± 0.09	0.33 ± 0.05

HC: healthy controls, PH: primary hypertension, PA: primary aldosteronism, LVEDVI: indexed left ventricle end diastolic volume; LVESVI: indexed left ventricle end systolic volume; LVMI: indexed left ventricle mass, M/V: mass/volume ratio; LVEF: left ventricle ejection fraction; LGE: late gadolinium enhancement; ECV: extracellular volume; ICV: intracellular volume; ECMI: indexed extracellular mass; ICMI: indexed intracellular mass.

^†^ p<0.05 PH versus HC,

^‡^ p<0.05 PA versus HC.

Near half of hypertensive patients presented similar small extent LGE, with a non-specific and non-subendocardial pattern, mostly in the insertion points of right and LV.

Remote and global myocardial native T1 and ECV did not statically differ across groups (p > 0.05 after adjustment for multiple comparisons). Hypertensive groups had similar ECMI and ICMI, that was significantly higher than controls.

**Fig 2:**
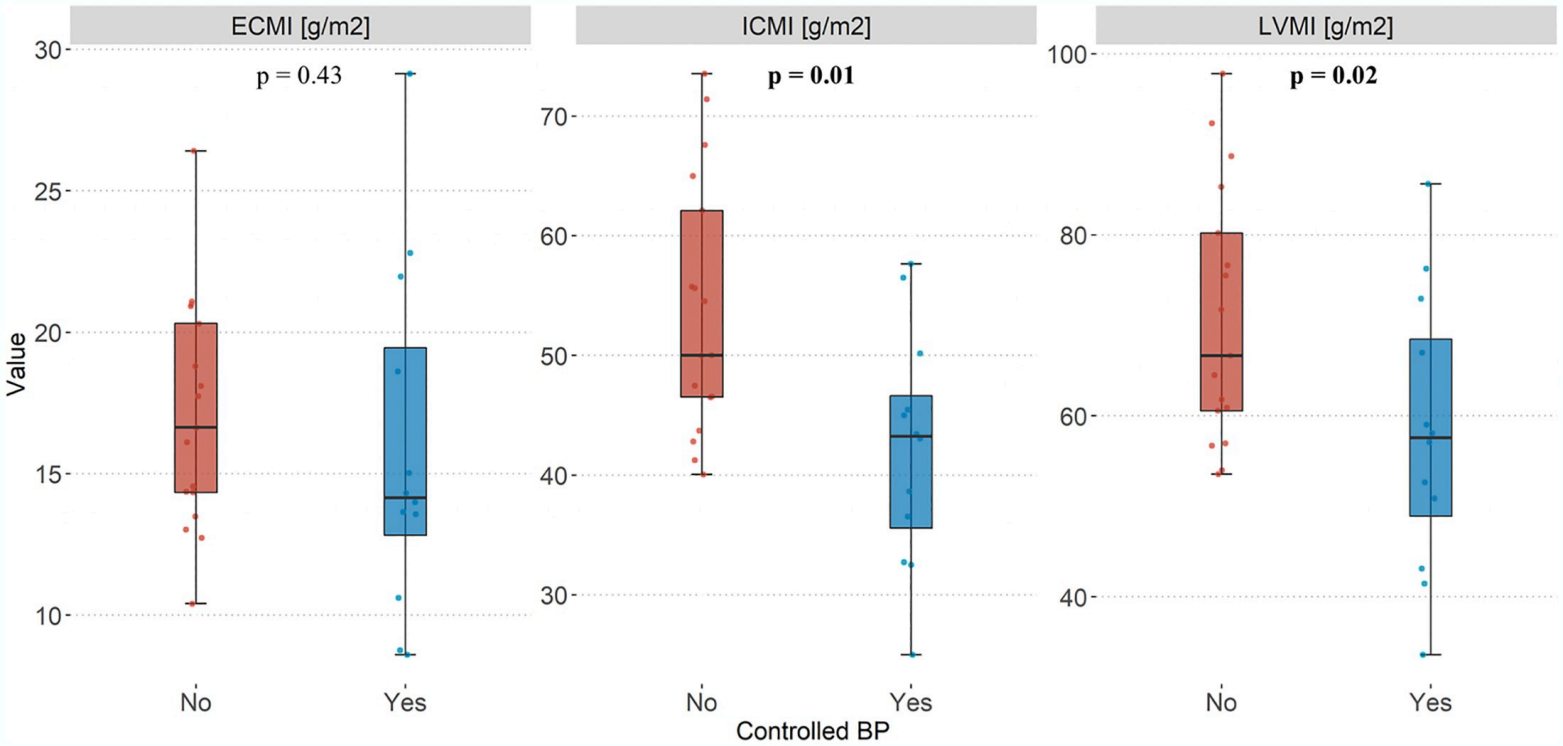
CMR T1 mapping derived parameters and blood pressure control across hypertensive groups. BP: blood pressure, ECMI: extracellular mass indexed, ICMI: intracellular mass index, LVMI: left ventricle mass indexed.

As expected, LVMI and ICMI was significantly lower in hypertensive patients who had controlled BP than those who did not (LVMI: 58 ± 15 g/m2 vs 72 ± 14g/m2, p = 0.02; ICMI: 43 ± 11 g/m2 vs 55 ± 11g/m2, p = 0.01) (Fig 2). These differences between controlled and non-controlled BP patients remained significant when adjusted for hypertensive etiology (Table 3).

**Table 3:** Adjusted Effects on LVMI and ICMI.

	LVMI	ICMI
	Estimate (p-value)	Estimate (p-value)
Controlled BP—Yes	-12.8 (p = 0.02)	-11.5 (0.009)
PA	5.77 (p = 0.29)	4.93 (0.23)

BP: blood pressure, PA: primary aldosteronism, LVMI: left ventricle mass indexed, ICMI: intracellular mass indexed.

**Fig 3:**
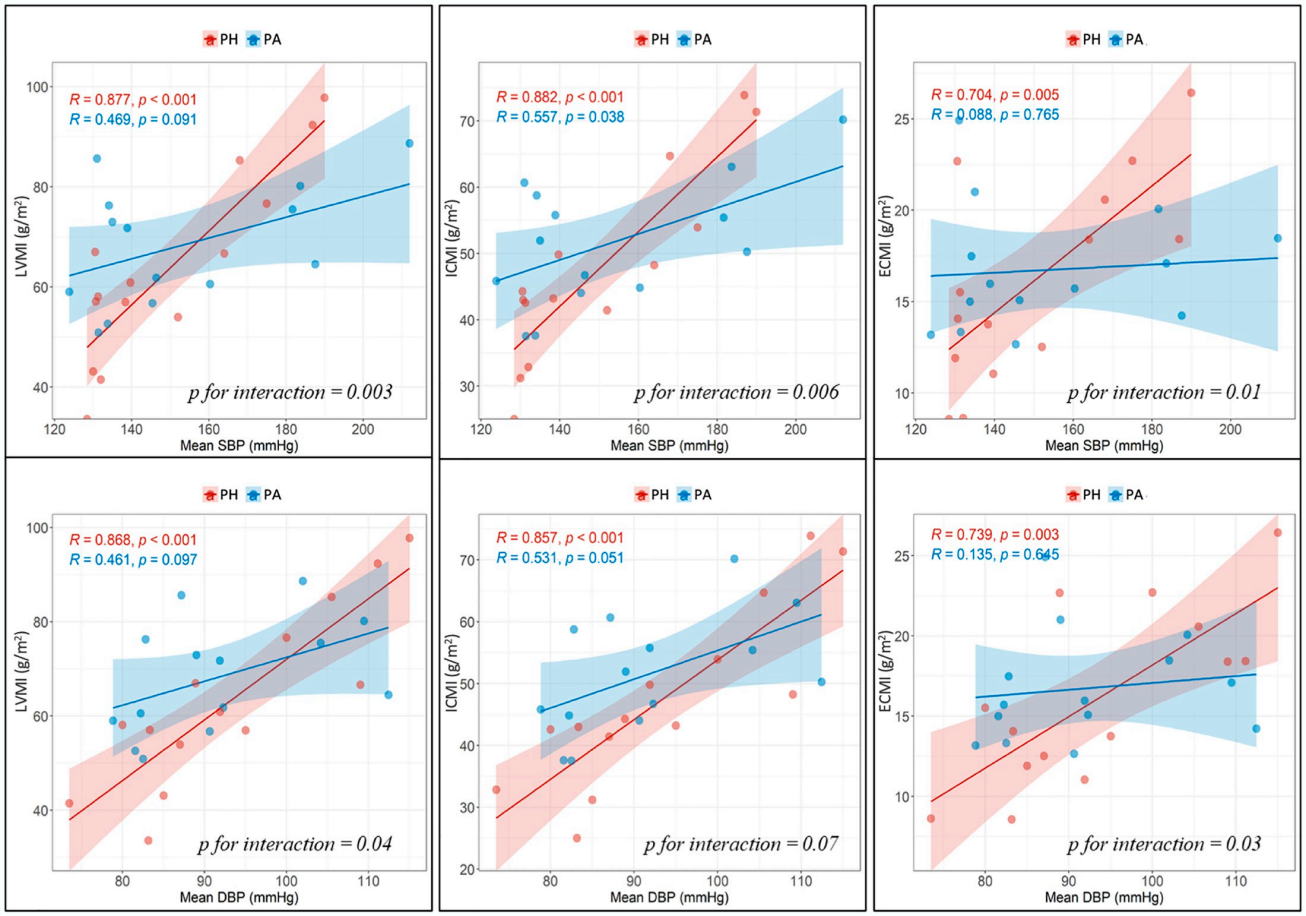
Correlation between CMR T1 mapping derived parameters and mean blood pressure in PH and PA patients, and their interaction by hypertension etiology. PH: primary hypertension, PA: primary aldosteronism, SBP: systolic blood pressure, DBP: diastolic blood pressure, ECMI: extracellular mass indexed, ICMI: intracellular mass index, LVMI: left ventricle mass indexed.

PH patients had strong correlations between mean BP measurements obtained during visits and LVMI, ICMI, and ECMI values (correlation coefficients ranging from 0.704 to 0.877, p<0.05 for all). These correlations were much weaker for PA patients, particularly SBP and DBP *vs* ECMI (0.088 and 0.135, p > 0.05 for both). There was also a significant interaction between hypertension etiology and BP measurements versus LVMI/ICMI/ECMI values (Fig 3).

## Discussion

We presented morphological, functional and tissue characterization by CMR of LVR in asymptomatic PH and PA patients, followed up and treated in a specialized tertiary unit. Our patients exhibited advanced hypertension, with high blood pressure levels at diagnosis and a long-term illness, near half of them exhibiting adequate blood pressure control at the time of CMR, despite a high number of drug classes prescribed in their medical records, including inhibitors of the RAAS. In this scenario, CMR showed similar residual subclinical tissue LVR between hypertensive groups, with an increase of the LVMI, at the expense of proportional increase in cardiomyocytes mass (ICMI) and interstitial matrix (ECMI), associated with a high prevalence of non-specific and non-subendocardial focal fibrosis. PA patients had worse geometric LVR, with higher prevalence of LVH and a tendency towards a higher M/V ratio (a marker of concentric remodeling). Unprecedented, to the best of our knowledge, we identified that the correlation between individual mean BP levels and the main LVR parameters of CMR (LVMI, ECMI and ICMI) was significantly modified by PA, whose positive correlations were weaker than those observed in PH, especially in the extracellular matrix.

In spite of the potential of CMR T1 mapping in LVR assessment, relatively few studies have investigated its role in patients with PA and PH. Since aldosterone excess has been linked to cardiomyocyte hypertrophy and LV fibrosis [8], it is expected to detect such changes with this method. In fact, prolonged native T1 times have been reported in PA patients in comparison to PH and/or normal individuals [20, 21], suggesting that aldosterone may in fact play a role in altering global myocardial tissue characteristics. However, studies that included ECV estimates have detected reduction [22], expantion [21] or no change [20]. Such discrepancies are probably related to populations differences, both in PA patients or in their paired controls, such as disease duration, use of anti-hypertensive treatment, blood pressure levels and presence of LVH. A proof-of-concept article [23] recently found no significant difference in native T1, ECV, and ICV values in PA patients using antihypertensive treatment not interfering with the RAAS (such as alpha-blockers, calcium channel blockers or central antihypertensives), in pair-wise analyses with PH individuals, and in normotensive patients with secondary hyperaldosteronism due to salt-spoiling tubulopathy (Bartter/Gitelman syndrome) and with healthy individuals. However, by indexing the T1 mapping parameters, they evidenced that increased ICMI was identified in both hypertensive groups, while expansion of the ECMI was evidenced only in the PA group, concluding that there is a synergic effect of aldosterone and high blood pressure on adverse LVR, especially in the extracellular matrix.

The lack of expansion in the ECMI of our PA patients sample in relation to PH controls can be attributed to some factors, isolated or in association, particular to our study: the greater prevalence of RAAS inhibitor medications in our patients at the time of CMR, which may have played a role in the prevention and/or reversal of interstitial expansion; the advanced disease of our PH control group, which may represent a population with more adverse tissue remodeling than that of previous studies; to our smaller sample size, which is a limitation of our work.

Reverse remodeling in the context of RAAS blockade is not an unknown fact [5, 24], including some evidence indicating that this occurs despite BP reductions. In animal models, the use of ACEi, ARB [25] and MRA [26] was able to reverse LVH and prevent the development of interstitial fibrosis. Studies with PH patients have shown that the administration of MRA associated with ACEi [27, 28], as well as the use of ARB [29], are related to the reversal of LVH, and pathological studies with ARB [30] have already shown regression of myocardial fibrosis, especially when more severe in baseline, which may have been the case in our cohort with a high prevalence of positive LGE. In PA individuals, administration of MRA has already shown to determine a significant reduction in LVMI calculated by CMR, detectable only three months after the institution of therapy, also associated with a significant diuretic effect, even in individuals with long-term disease [31]. This relatively rapid change in LVMI after aldosterone block raises the hypothesis that it may be, at least in part, related to the reversal of myocardial interstitial edema, as already observed in an ultrastructural histological study after adenomectomy [32]. This finding may help to explain why LVH in patients with familial PA precedes the increase in BP [33], being also more exacerbated and/or prevalent than PH individuals despite the similar blood pressure level [34], and, mainly, dependent on a high-sodium diet [35, 36].

Previous studies demonstrated that expansion of ECV and ECMI is more noticeable in PH patients with LVH and high BP levels [37–39], however, it’s not clear if there would be a difference in these parameters between PH without LVH and normotensive controls, since this was not explored by the authors [39]. In this context, the high prevalence of LGE in our PH patients seems to confirm that they represent a population with advanced disease, as they are more in line with those found by Rudolph et al [40] in PH with LVH, being higher than those reported in cohorts that compared them to PA [41].

As for our small cohort, the difference obtained in the quantification of the ECMI between the EH and PA groups was small and, if maintained, should remain with low significance when increasing the sample size. It is interesting to observe that, even with such a restricted sample, we observed a good positive correlation between median BP levels obtained and worse LVR parameters, significantly modified by PA. Such correlation between BP level, native T1 and ECV has already been detected in PH patients, albeit weaker [39]. In studies with other causes of left ventricular overload, such as aortic stenosis [42, 43], indexed T1 mapping parameters showed better discrimination between cases and controls, as well as good positive correlation with the increase in the degree of severity of the disease, which may be analogous to the insult offered by sustained exposure to different blood pressure levels in hypertension context.

It is quite relevant that even with specialized monitoring and the prescribed treatment, just under half of our hypertensive individuals effectively achieved BP control, a prevalence slightly above the average previously reported in population surveys in our continent (36.2%) [3]. The remainder of our cohort could be classified as “apparently” resistant hypertension [18], since BP control was not achieved despite treatment (87% of these patients were prescribed 3 or more classes of antihypertensive drugs). The fact that this study was not designed to be a controlled clinical trial is a limitation, however, our findings represent the real-life scenario, where physicians who prescribe antihypertensive interventions to ensure BP control, even in specialized units, may not succeed in preventing adverse outcomes if there is resistance to a more aggressive approach or reduced patient awareness on hypertensive disease [4].

In clinical trials with emphasis on BP control, renal denervation treatment in 12 individuals with drug-resistant PH revealed that decreasing BP was positively correlated with reduction in ECV six months after the procedure, however, no changes in native T1 or in LVMI were detected [44]. In a substudy with CMR of the SPRINT trial [45], there was no statistically significant change in LVMI, systolic function and native T1 among patients treated for aggressive BP control *versus* those with standard BP control. The lack of prognostic impact on the reversal of LVR parameters measured by CMR may, initially, seem antagonistic to previous evidence that correlates reduction of LVMI with reduction in cardiovascular risk independently of BP control [46, 47]. However, these statements are not mutually exclusive. Notwithstanding, controlling BP does not seems to fully reverse LVR [5], which is multifactorial, but our findings indicate that uncontrolled BP is correlated with persistence of adverse LVR, specially increased LVMI and cardiomyocyte hypertrophy, regardless of hypertension etiology. In parallel, they reinforce the greater complexity of adverse LVR in treated PA individuals, where BP levels seem to play a more marginal role, especially in the extracellular matrix.

In summary, CMR proves to be a non-invasive method with incremental value by adding information on compartmental remodeling of myocardial tissue. This ability defies previously accepted concepts about LVR in increased afterload conditions, especially those encompassed under the “hypertrophy” label, since increased mass or thickness may reflect different compartmental responses to the same insult [48]. Currently, this surpasses the understanding of its prognostic impact, especially in the context of patients with resistant hypertension [10, 23]. As knowledge expands towards therapies aimed at targeting individual components of LVR [5], we hope that even small studies such as this one can help highlight the potential of CMR as a robust cardiac biomarker [49], able to assess disease progression and its response to treatment.

### Limitations

As an underdiagnosed disease, this relatively small sample of patients with PA may not have representatively contemplated all its etiologies and presentations of geometric remodeling. The small sample size may limit the detection of minor changes in myocardial remodeling, particularly for parameters at the detection threshold of current CMR techniques. A greater number of patients was not reached since the COVID-19 pandemic limited the performance of CMR exams for research purposes in the final acquisition phase of our cohort. Also, hematocrit levels were not obtained on the same day as the CMR acquisition for all patients. Nonetheless, evidence supports the use of off-day hematocrit measurements as reliable for the calculation of ECV [50]. Besides, native and post-contrast T1 times (independent variables from hematocrit) did not differ between the three groups studied, reinforcing this might not be a major reason for the absence of statistical difference in our cohort. Unfortunately, T2 mapping of the left ventricle was not obtained during imaging acquisition. Moreover, since our study focus was on left ventricle remodeling, right ventricular volumes, ejection fraction, left atrial area, and LV longitudinal strain were not included in our analysis.

The potential influence of medications on myocardial remodeling markers could not be fully evaluated due to the limited sample size. Future studies with larger cohorts are needed to assess the interaction between antihypertensive drugs and myocardial remodeling markers to provide more comprehensive insights.

Treatment-naïve patients are rare in our tertiary specialized hypertensive unit and, therefore, an additional control group was not included. Also, adherence to the treatment prescribed in medical records and dose optimization were not accessed. Ambulatory and home BP monitoring were not obtained in our hypertensive groups data, thus, measurement bias, especially related to the “white coat” and masked effects, may not have been considered [1]. Since this is a cross-sectional case-control study, it is not possible to establish a causal relationship between the lack of BP control and cardiomyocyte hypertrophy, or the blockade of RAAS and the protective effect on adverse ECMI remodeling.

## References

[1] Whelton PK, Carey RM, Aronow WS, Casey DE, Collins KJ, Himmelfarb CD, et al. 2017 ACC/AHA/AAPA/ABC/ACPM/AGS/APhA/ ASH/ASPC/NMA/PCNA guideline for the prevention, detection, evaluation, and management of high blood pressure in adults a report of the American College of Cardiology/American Heart Association Task Force on Clinical practice guidelines. Hypertension. 2018 Jun 1;71(6):E13–115. doi: 10.1161/HYP.0000000000000065 29133356

[2] Banegas JR, López-García E, Dallongeville J, Guallar E, Halcox JP, Borghi C, et al. Achievement of treatment goals for primary prevention of cardiovascular disease in clinical practice across Europe: the EURIKA study. Eur Heart J [Internet]. 2011 Sep [cited 2022 Mar 11];32(17):2143–52. Available from: https://pubmed.ncbi.nlm.nih.gov/21471134/. doi: 10.1093/eurheartj/ehr080 21471134 PMC3164103

[3] Chow CK, Teo KK, Rangarajan S, Islam S, Gupta R, Avezum A, et al. Prevalence, awareness, treatment, and control of hypertension in rural and urban communities in high-, middle-, and low-income countries. JAMA [Internet]. 2013 Sep 4 [cited 2022 Mar 11];310(9):959–68. Available from: https://pubmed.ncbi.nlm.nih.gov/24002282/. doi: 10.1001/jama.2013.184182 24002282

[4] Muntner P, Hardy ST, Fine LJ, Jaeger BC, Wozniak G, Levitan EB, et al. Trends in blood pressure control among US adults with hypertension, 1999–2000 to 2017–2018. JAMA—Journal of the American Medical Association. 2020 Sep 22;324(12):1190–200. doi: 10.1001/jama.2020.14545 32902588 PMC7489367

[5] González A, Ravassa S, López B, Moreno MU, Beaumont J, San José G, et al. Myocardial remodeling in hypertension toward a new view of hypertensive heart disease. Hypertension [Internet]. 2018 [cited 2022 Mar 7];72(3):549–58. Available from:https://www.ahajournals.org/doi/suppl/10.1161/HYPERTENSIONAHA.118.11125. 30354762 10.1161/HYPERTENSIONAHA.118.11125

[6] Vakili BA, Okin PM, Devereux RB. Prognostic implications of left ventricular hypertrophy. Am Heart J. 2001 Mar 1;141(3):334–41. doi: 10.1067/mhj.2001.113218 11231428

[7] Gjesdal O, Bluemke DA, Lima JA. Cardiac remodeling at the population level—risk factors, screening, and outcomes. Nat Rev Cardiol [Internet]. 2011 Dec [cited 2022 Mar 18];8(12):673–85. Available from: https://pubmed.ncbi.nlm.nih.gov/22027657/. doi: 10.1038/nrcardio.2011.154 22027657

[8] Tsai CH, Pan Chien-Ting •, Chang YY, Chen ZW, Wu VC, Hung CS, et al. Left ventricular remodeling and dysfunction in primary aldosteronism. J Hum Hypertens [Internet]. 2021 [cited 2022 Mar 7];35:131–47. Available from: 10.1038/s41371-020-00426-y. 33067554 PMC7895762

[9] Gregory Hundley W, Bluemke DA, Paul Finn J, Flamm SD, Fogel MA, Friedrich MG, et al. ACCF/ACR/AHA/NASCI/SCMR 2010 Expert Consensus Document on Cardiovascular Magnetic Resonance ACCF Task Force Members. JAC [Internet]. 2010 [cited 2022 Mar 10];55(23):2614–62. Available from: www.acc.org. 10.1016/j.jacc.2009.11.011 PMC3042771 20513610

[10] Tadic M, Cuspidi C, Plein S, Milivojevic IG, Wang DW, Grassi G, et al. Comprehensive assessment of hypertensive heart disease: cardiac magnetic resonance in focus. Heart Fail Rev [Internet]. 2021 Nov 1 [cited 2022 Mar 10];26(6):1383–90. Available from: https://pubmed.ncbi.nlm.nih.gov/32170529/. doi: 10.1007/s10741-020-09943-x 32170529

[11] Messroghli DR, Moon JC, Ferreira VM, Grosse-Wortmann L, He T, Kellman P, et al. Clinical recommendations for cardiovascular magnetic resonance mapping of T1, T2, T2* and extracellular volume: A consensus statement by the Society for Cardiovascular Magnetic Resonance (SCMR) endorsed by the European Association for Cardiovascular Imaging (EACVI). Journal of Cardiovascular Magnetic Resonance 2017 19:1 [Internet]. 2017 Oct 9 [cited 2022 Mar 11];19(1):1–24. Available from: https://jcmr-online.biomedcentral.com/articles/10.1186/s12968-017-0389-8. 10.1186/s12968-017-0389-8 PMC5633041 28992817

[12] Brandão AA, Rodrigues CIS, Consolim-Colombo F, Plavnik FL, Malachias MVB, Kohlmann O, et al. VI Diretrizes Brasileiras de Hipertensão. Arq Bras Cardiol [Internet]. 2010 Jul [cited 2024 Oct 30];95(1 Suppl):I–III. Available from: https://www.scielo.br/j/abc/a/Q43xYKS4fJsRM8jj8s4pxSJ/.

[13] Araujo-Filho JAB, Assuncao AN, Tavares De Melo MD, Bière L, Lima CR, et al. Myocardial T1 mapping and extracellular volume quantification in patients with left ventricular non-compaction cardiomyopathy. Eur Heart J Cardiovasc Imaging [Internet]. 2018 Aug 1 [cited 2024 Sep 2];19(8):888–95. Available from: https://pubmed.ncbi.nlm.nih.gov/29518212/. doi: 10.1093/ehjci/jey022 29518212

[14] Melo RJL, Assunção AN, Morais TC, Nomura CH, Scanavacca MI, Martinelli-Filho M, et al. Detection of Early Diffuse Myocardial Fibrosis and Inflammation in Chagas Cardiomyopathy with T1 Mapping and Extracellular Volume. Radiol Cardiothorac Imaging [Internet]. 2023 Jun 1 [cited 2024 Sep 2];5(3). Available from: https://pubs.rsna.org/doi/10.1148/ryct.220112. 37404789 10.1148/ryct.220112 PMC10316290

[15] Kramer CM, Barkhausen J, Bucciarelli-Ducci C, Flamm SD, Kim RJ, Nagel E. Standardized cardiovascular magnetic resonance imaging (CMR) protocols: 2020 update. J Cardiovasc Magn Reson [Internet]. 2020 Feb 24 [cited 2022 Mar 11];22(1). Available from: https://pubmed.ncbi.nlm.nih.gov/32089132/. doi: 10.1186/s12968-020-00607-1 PMC7038611 32089132

[16] Schulz-Menger J, Bluemke DA, Bremerich J, Flamm SD, Fogel MA, Friedrich MG, et al. Standardized image interpretation and post-processing in cardiovascular magnetic resonance—2020 update: Society for Cardiovascular Magnetic Resonance (SCMR): Board of Trustees Task Force on Standardized Post-Processing. J Cardiovasc Magn Reson [Internet]. 2020 Mar 12 [cited 2022 Mar 11];22(1). Available from: https://pubmed.ncbi.nlm.nih.gov/32160925/. doi: 10.1186/s12968-020-00610-6 PMC7066763 32160925

[17] Olivotto I, Maron MS, Autore C, Lesser JR, Rega L, Casolo G, et al. Assessment and significance of left ventricular mass by cardiovascular magnetic resonance in hypertrophic cardiomyopathy. J Am Coll Cardiol [Internet]. 2008 Aug 12 [cited 2022 Mar 11];52(7):559–66. Available from: https://pubmed.ncbi.nlm.nih.gov/18687251/. doi: 10.1016/j.jacc.2008.04.047 18687251

[18] Egan BM, Zhao Y, Axon RN, Brzezinski WA, Ferdinand KC. Uncontrolled and apparent treatment resistant hypertension in the United States, 1988 to 2008. Circulation [Internet]. 2011 Aug 30 [cited 2022 Mar 19];124(9):1046–58. Available from: http://circ.ahajournals.org. doi: 10.1161/CIRCULATIONAHA.111.030189 21824920 PMC3210066

[19] Hommel G. A stagewise rejective multiple test procedure based on a modified Bonferroni test. Biometrika [Internet]. 1988 Jun 1 [cited 2022 Mar 16];75(2):383–6. Available from: https://academic.oup.com/biomet/article/75/2/383/292949.

[20] Wu T, Ren Y, Wang W, Cheng W, Zhou F, et al. Left Ventricular Remodeling in Patients with Primary Aldosteronism: A Prospective Cardiac Magnetic Resonance Imaging Study. Korean J Radiol [Internet]. 2021 Oct 1 [cited 2022 Mar 10];22(10):1619–27. Available from: https://pubmed.ncbi.nlm.nih.gov/34269528/. doi: 10.3348/kjr.2020.1291 34269528 PMC8484156

[21] Zhou F, Wu T, Wang W, Cheng W, Wan S, Tian H, et al. CMR-Verified Myocardial Fibrosis Is Associated With Subclinical Diastolic Dysfunction in Primary Aldosteronism Patients. Front Endocrinol (Lausanne) [Internet]. 2021 May 14 [cited 2022 Mar 10];12. Available from: https://pubmed.ncbi.nlm.nih.gov/34054733/. doi: 10.3389/fendo.2021.672557 PMC8160454 34054733

[22] Grytaas MA, Sellevåg K, Thordarson HB, Husebye ES, Løvås K, Larsen TH. Cardiac magnetic resonance imaging of myocardial mass and fibrosis in primary aldosteronism. 2018; Available from: 10.1530/EC-18-0039. PMC5834771 29440130

[23] Redheuil A, Blanchard A, Pereira H, Raissouni Z, Lorthioir A, Soulat G, et al. Aldosterone-Related Myocardial Extracellular Matrix Expansion in Hypertension in Humans: A Proof-of-Concept Study by Cardiac Magnetic Resonance. JACC Cardiovasc Imaging [Internet]. 2020 Oct 1 [cited 2022 Mar 7];13(10):2149–59. Available from: 10.1016/j.jcmg.2020.06.026. 32950448

[24] Fagard RH, Celis H, Thijs L, Wouters S. Heart Regression of Left Ventricular Mass by Antihypertensive Treatment A Meta-Analysis of Randomized Comparative Studies. 2009; http://ahajournals.org. 10.1161/HYPERTENSIONAHA.109.136655 19770405

[25] Tamura T, Said S, Harris J, Lu W, Gerdes AM. Reverse remodeling of cardiac myocyte hypertrophy in hypertension and failure by targeting of the renin-angiotensin system. Circulation [Internet]. 2000 Jul 11 [cited 2022 Apr 1];102(2):253–9. Available from: https://pubmed.ncbi.nlm.nih.gov/10889139/ doi: 10.1161/01.cir.102.2.253 10889139

[26] Coelho-Filho OR, Shah R v., Neilan TG, Mitchell R, Moreno H, Kwong R, et al. Cardiac magnetic resonance assessment of interstitial myocardial fibrosis and cardiomyocyte hypertrophy in hypertensive mice treated with spironolactone. J Am Heart Assoc [Internet]. 2014 [cited 2022 Apr 1];3(3). Available from: https://pubmed.ncbi.nlm.nih.gov/24965024/. doi: 10.1161/JAHA.114.000790 PMC4309062 24965024

[27] Sato A, Hayashi M, Saruta T. Relative long-term effects of spironolactone in conjunction with an angiotensin-converting enzyme inhibitor on left ventricular mass and diastolic function in patients with essential hypertension. Hypertens Res [Internet]. 2002 [cited 2022 Apr 1];25(6):837–42. Available from: https://pubmed.ncbi.nlm.nih.gov/12484506/. doi: 10.1291/hypres.25.837 12484506

[28] Pitt B, Reichek N, Willenbrock R, Zannad F, Phillips RA, Roniker B, et al. Effects of eplerenone, enalapril, and eplerenone/enalapril in patients with essential hypertension and left ventricular hypertrophy: the 4E-left ventricular hypertrophy study. Circulation [Internet]. 2003 Oct 14 [cited 2022 Apr 1];108(15):1831–8. Available from: https://pubmed.ncbi.nlm.nih.gov/14517164/. doi: 10.1161/01.CIR.0000091405.00772.6E 14517164

[29] Devereux RB, Dahlöf B, Gerdts E, Boman K, Nieminen MS, Papademetriou V, et al. Regression of hypertensive left ventricular hypertrophy by losartan compared with atenolol: the Losartan Intervention for Endpoint Reduction in Hypertension (LIFE) trial. Circulation [Internet]. 2004 Sep 14 [cited 2022 Apr 8];110(11):1456–62. Available from: https://pubmed.ncbi.nlm.nih.gov/15326072/. doi: 10.1161/01.CIR.0000141573.44737.5A 15326072

[30] Díez J, Querejeta R, López B, González A, Larman M, Martínez Ubago JL. Losartan-dependent regression of myocardial fibrosis is associated with reduction of left ventricular chamber stiffness in hypertensive patients. Circulation [Internet]. 2002 May 28 [cited 2022 Apr 6];105(21):2512–7. Available from: https://pubmed.ncbi.nlm.nih.gov/12034658/. doi: 10.1161/01.cir.0000017264.66561.3d 12034658

[31] Gaddam K, Corros C, Pimenta E, Ahmed M, Denney T, Aban I, et al. Heart Rapid Reversal of Left Ventricular Hypertrophy and Intracardiac Volume Overload in Patients With Resistant Hypertension and Hyperaldosteronism A Prospective Clinical Study From the Division of Cardiovascular Disease, Departments of Medicine (K. 2010; http://ahajournals.org. 10.1161/HYPERTENSIONAHA.109.141531 PMC2864599 20351345

[32] Frustaci A, Letizia C, Verardo R, Grande C, Francone M, Sansone L, et al. Primary aldosteronism-associated cardiomyopathy: Clinical-pathologic impact of aldosterone normalization. Int J Cardiol. 2019 Oct 1;292:141–7. doi: 10.1016/j.ijcard.2019.06.055 31256994

[33] Stowasser M, Sharman J, Leano R, Gordon RD, Ward G, Cowley D, et al. Evidence for abnormal left ventricular structure and function in normotensive individuals with familial hyperaldosteronism type I. J Clin Endocrinol Metab [Internet]. 2005 Sep [cited 2022 Apr 23];90(9):5070–6. Available from: https://pubmed.ncbi.nlm.nih.gov/15941863/. doi: 10.1210/jc.2005-0681 15941863

[34] Rossi GP, Sacchetto A, Pavan E, Palatini P, Graniero GR, Canali C, et al. Remodeling of the left ventricle in primary aldosteronism due to Conn’s adenoma. Circulation [Internet]. 1997 [cited 2022 Apr 23];95(6):1471–8. Available from: https://pubmed.ncbi.nlm.nih.gov/9118515/. doi: 10.1161/01.cir.95.6.1471 9118515

[35] Pimenta E, Gordon RD, Stowasser M. Salt, aldosterone and hypertension. J Hum Hypertens [Internet]. 2013 Jan [cited 2022 Apr 23];27(1):1–6. Available from: https://pubmed.ncbi.nlm.nih.gov/22785050/. doi: 10.1038/jhh.2012.27 22785050

[36] Catena C, Colussi GL, Novello M, Verheyen ND, Bertin N, Pilz S, et al. Dietary Salt Intake Is a Determinant of Cardiac Changes After Treatment of Primary Aldosteronism: A Prospective Study. Hypertension [Internet]. 2016 Jul 1 [cited 2022 Apr 23];68(1):204–12. Available from: https://pubmed.ncbi.nlm.nih.gov/27245179/. doi: 10.1161/HYPERTENSIONAHA.116.07615 27245179

[37] Treibel TA, Zemrak F, Sado DM, Banypersad SM, White SK, Maestrini V, et al. Extracellular volume quantification in isolated hypertension—changes at the detectable limits? J Cardiovasc Magn Reson [Internet]. 2015 Aug 12 [cited 2022 Apr 7];17(1). Available from: https://pubmed.ncbi.nlm.nih.gov/26264919/. doi: 10.1186/s12968-015-0176-3 PMC4534050 26264919

[38] Kuruvilla S, Janardhanan R, Antkowiak P, Keeley EC, Adenaw N, Brooks J, et al. Increased extracellular volume and altered mechanics are associated with LVH in hypertensive heart disease, not hypertension alone. JACC Cardiovasc Imaging [Internet]. 2015 Feb 1 [cited 2022 Apr 6];8(2):172–80. Available from: https://pubmed.ncbi.nlm.nih.gov/25577446/. doi: 10.1016/j.jcmg.2014.09.020 25577446 PMC4418794

[39] Rodrigues JCL, Amadu AM, Dastidar AG, Szantho G v., Lyen SM, Godsave C, et al. Comprehensive characterisation of hypertensive heart disease left ventricular phenotypes. Heart [Internet]. 2016 Oct 15 [cited 2022 Apr 6];102(20):1671–9. Available from: https://pubmed.ncbi.nlm.nih.gov/27260191/. doi: 10.1136/heartjnl-2016-309576 27260191 PMC5099214

[40] Rudolph A, Abdel-Aty H, Bohl S, Boyé P, Zagrosek A, Dietz R, et al. Noninvasive detection of fibrosis applying contrast-enhanced cardiac magnetic resonance in different forms of left ventricular hypertrophy relation to remodeling. J Am Coll Cardiol [Internet]. 2009 Jan 20 [cited 2022 Apr 7];53(3):284–91. Available from: https://pubmed.ncbi.nlm.nih.gov/19147047/. doi: 10.1016/j.jacc.2008.08.064 19147047

[41] Freel EM, Mark PB, Weir RAP, McQuarrie EP, Allan K, Dargie HJ, et al. Demonstration of blood pressure-independent noninfarct myocardial fibrosis in primary aldosteronism: a cardiac magnetic resonance imaging study. Circ Cardiovasc Imaging [Internet]. 2012 Nov [cited 2022 Apr 5];5(6):740–7. Available from: https://pubmed.ncbi.nlm.nih.gov/23019275/. doi: 10.1161/CIRCIMAGING.112.974576 23019275

[42] Chin CWL, Everett RJ, Kwiecinski J, Vesey AT, Yeung E, Esson G, et al. Myocardial Fibrosis and Cardiac Decompensation in Aortic Stenosis. JACC Cardiovasc Imaging [Internet]. 2017 Nov 1 [cited 2022 Apr 6];10(11):1320–33. Available from: https://pubmed.ncbi.nlm.nih.gov/28017384/. doi: 10.1016/j.jcmg.2016.10.007 28017384 PMC5683736

[43] Everett RJ, Tastet L, Clavel MA, Chin CWL, Capoulade R, Vassiliou VS, et al. Progression of hypertrophy and myocardial fibrosis in aortic stenosis: A multicenter cardiac magnetic resonance study. Circ Cardiovasc Imaging [Internet]. 2018 Jun 1 [cited 2022 Apr 6];11(6):e007451. Available from: /pmc/articles/PMC6023592/. doi: 10.1161/CIRCIMAGING.117.007451 29914867 PMC6023592

[44] Delacroix S, Chokka RG, Nelson AJ, Wong DT, Pederson S, Nimmo J, et al. Effects of renal sympathetic denervation on myocardial structure, function and perfusion: A serial CMR study. Atherosclerosis [Internet]. 2018 May 1 [cited 2022 Apr 7];272:207–15. Available from: https://pubmed.ncbi.nlm.nih.gov/29627741/. doi: 10.1016/j.atherosclerosis.2018.03.022 29627741

[45] Upadhya B, Rocco M v., Pajewski NM, Morgan T, Blackshear J, Hundley WG, et al. Effect of Intensive Blood Pressure Reduction on Left Ventricular Mass, Structure, Function, and Fibrosis in the SPRINT-HEART. Hypertension [Internet]. 2019 Aug 1 [cited 2022 Apr 2];74(2):276–84. Available from: https://biolincc.nhlbi.nih.gov/. doi: 10.1161/HYPERTENSIONAHA.119.13073 31256724 PMC7098010

[46] Verdecchia P, Schillaci G, Borgioni C, Ciucci A, Gattobigio R, Zampi I, et al. Prognostic Significance of Serial Changes in Left Ventricular Mass in Essential Hypertension. Circulation [Internet]. 1998 Jan 13 [cited 2022 Mar 18];97(1):48–54. Available from: https://www.ahajournals.org/doi/abs/10.1161/01.cir.97.1.48. 9443431 10.1161/01.cir.97.1.48

[47] Devereux RB, Wachtell K, Gerdts E, Boman K, Nieminen MS, Papademetriou V, et al. Prognostic Significance of Left Ventricular Mass Change During Treatment of Hypertension. JAMA [Internet]. 2004 Nov 17 [cited 2022 Mar 18];292(19):2350–6. Available from: https://jamanetwork.com/journals/jama/fullarticle/199809. doi: 10.1001/jama.292.19.2350 15547162

[48] Knöll R, Iaccarino G, Tarone G, Hilfiker-Kleiner D, Bauersachs J, Leite-Moreira AF, et al. Towards a re-definition of “cardiac hypertrophy” through a rational characterization of left ventricular phenotypes: a position paper of the Working Group “Myocardial Function” of the ESC. Eur J Heart Fail [Internet]. 2011 Aug [cited 2022 Mar 27];13(8):811–9. Available from: https://pubmed.ncbi.nlm.nih.gov/21708908/. doi: 10.1093/eurjhf/hfr071 21708908

[49] Taylor AJ, Gutman SJ. Myocardial T1 Mapping in Heart Disease: Research Tool or New Cardiac Biomarker? JACC Cardiovasc Imaging. 2020 Jan 1;13(1):55–7. doi: 10.1016/j.jcmg.2019.05.003 31202764

[50] Su MY, Huang Y Sen, Niisato E, Chow K, JMJ, Wu CK, et al. Is a timely assessment of the hematocrit necessary for cardiovascular magnetic resonance—derived extracellular volume measurements? Journal of Cardiovascular Magnetic Resonance [Internet]. 2020 Dec 1 [cited 2024 Nov 1];22(1):1–11. Available from: https://jcmr-online.biomedcentral.com/articles/10.1186/s12968-020-00689-x 33250055 10.1186/s12968-020-00689-x PMC7702722

